# Differentially expressed genes in cotyledon of ewes fed mycotoxins

**DOI:** 10.1186/s12864-020-07074-z

**Published:** 2020-10-01

**Authors:** J. L. Britt, R. E. Noorai, S. K. Duckett

**Affiliations:** 1 Department of Animal and Veterinary Sciences, Clemson University, Clemson, SC 29634 USA; 2 Clemson University Genomics and Bioinformatics Facility, Clemson University, Clemson, SC 29634 USA

**Keywords:** Sheep, Ergot alkaloid, Mycotoxin, Cotyledon, Vasoconstriction, Nutrient transport

## Abstract

**Background:**

Ergot alkaloids (E+) are mycotoxins produced by the endophytic fungus, *Epichloë coenophiala,* in tall fescue that are associated with ergotism in animals. Exposure to ergot alkaloids during gestation reduces fetal weight and placental mass in sheep. These reductions are related to vasoconstrictive effects of ergot alkaloids and potential alterations in nutrient transport to the fetus. Cotyledon samples were obtained from eight ewes that were fed E+ (*n* = 4; E+/E+) or E- (endophyte-free without ergot alkaloids; n = 4; E−/E-) seed during both mid (d 35 to 85) and late (d 85–133) gestation to assess differentially expressed genes associated with ergot alkaloid induced reductions in placental mass and fetal weight, and discover potential adaptive mechanisms to alter nutrient supply to fetus.

**Results:**

Ewes fed E+/E+ fescue seed during both mid and late gestation had 20% reduction in fetal body weight and 33% reduction in cotyledon mass compared to controls (E−/E-). Over 13,000 genes were identified with 110 upregulated and 33 downregulated. Four genes had a |log2FC| > 5 for ewes consuming E+/E+ treatment compared to controls: *LECT2*, *SLC22A9*, *APOC3*, and *MBL2*. REViGO revealed clusters of upregulated genes associated glucose, carbohydrates, lipid, protein, macromolecular and cellular metabolism, regulation of wound healing and response to starvation. For downregulated genes, no clusters were present, but all enriched GO terms were associated with anion and monocarboxylic acid transport. The complement and coagulation cascade and the peroxisome proliferator-activated receptor signaling pathway were found to be enriched for ewes consuming E+/E+ treatment.

**Conclusions:**

Consumption of ergot alkaloids during gestation altered the cotyledonary transcriptome specifically related to macronutrient metabolism, wound healing and starvation. These results show that ergot alkaloid exposure upregulates genes involved in nutrient metabolism to supply the fetus with additional substrates in attempts to rescue fetal growth.

## Background

Tall fescue [*Lolium arundinaceum* (Schreb.) Darbysh; *Schedonorus phoenix* (Scop.) Holub] is the primary cool season perennial grass utilized in the eastern U.S. occupying more than 14 million ha [1]. The majority of tall fescue contains an endophyte (*Epichloë coenophiala*), which produces ergot alkaloids (i.e. ergovaline, ergovalinine, etc. [2];, mycotoxins associated with ergotism in humans and animals. The endophyte is beneficial to the plant and aids in establishment, persistence, and drought tolerance [1]; however, ingestion of ergot alkaloids by grazing livestock results in fescue toxicosis, a syndrome which reduces animal growth [3] and reproductive performance [4]. Previous research shows that exposure to ergovaline and ergovalinine during late (d 86 to parturition) gestation alters placental development [5, 6] and vasoactivity of umbilical arteries [7], and results in asymmetrical growth and intrauterine growth restriction of the fetus [8]; however, little is known about the mechanisms associated with these changes in the placenta.

Intrauterine growth restriction (IUGR) is the inability of a fetus to reach its expected growth potential in-utero, which leads to 10-fold higher rates of morbidity and mortality, the primary cause of stillbirth in humans [9–11]. IUGR is characterized by asymmetrical growth in which brain development is spared at the expense of overall growth [12]. The most common cause of IUGR is placental insufficiency where reductions in placental mass or impaired placental transport capacity lead to an inefficient transfer of substrates to the growing fetus [13]. In most mammalian species, uterine and umbilical blood flows are directly correlated with placental and fetal weights [14, 15] and reductions in uterine blood flow can lead to stunted fetal growth and an increase in offspring morbidity and mortality [16, 17]. Conditions that negatively influence fetal growth, such as maternal undernutrition or increased fetal number, are often associated with reductions in nutrient uptake, blood flow, and placental angiogenesis, the formation of new vascular beds [18, 19]. Angiogenesis and vasodilation of the uterine and placental vessels are primary mechanisms that work to increase blood flow during gestation. Furthermore, establishment of efficient fetal and maternal vascular beds within the placenta is crucial to support the rapid increase in placental blood flow, especially during the later stages of gestation when the majority of fetal growth occurs [14, 15].

Placental insufficiency and subsequent IUGR can be induced in gestating sheep through the consumption of ergovaline, an ergot alkaloid found in endophyte-infected tall fescue known to cause systematic vasoconstriction [6, 8]. Ergovaline binds to 5-hydroxytryptamine receptor 2A (5HT2a) and α-2 adrenergic receptors located throughout the cardiovascular system. These receptors are also located in the umbilical and uterine arteries of pregnant sheep and cows [20–22]. Vasoconstrictive activity associated with ergot alkaloid exposure has also been reported in the uterine vessels of heifers, uterine arteries of pregnant sheep, and umbilical arteries in sheep [7, 20, 23]. The resulting vasoconstriction reduces uteroplacental blood flow and limits fetal growth [6]. Sheep are utilized as a model for placental insufficiency and IUGR in humans due to similarities in placental structure and parallel IUGR pathologies after birth and into adulthood [24–27]. Proper placental vascularization and function depends on the finely tuned regulation of gene expression throughout gestation [28]. Aberrant gene expression in the placenta indicates altered molecular pathways which can provide insight into the causes of diseases such as preeclampsia and IUGR. This makes RNA-sequencing useful in detecting altered pathways associated with diseases states during pregnancy [29, 30]. It was hypothesized that reduction in placental and fetal mass was likely due to the vasoconstrictive effects exerted on uterine and placental blood flow that are commonly associated with ergot alkaloid exposure [20]. Several known angiogenic and vascular growth factors were investigated in the cotyledon tissues of ergovaline-fed ewes, but no differences were found that could account for the overall reduction in placental mass [6]. Therefore, the objective of this study was to assess differentially expressed genes using RNA-Seq associated with ergot alkaloid induced reductions in placentome and fetal weight and discover potential adaptive mechanisms to alter nutrient supply to fetus.

## Results

### Placentomes

Total placentome weight was 30% lower (*P* < 0.05) for E+/E+ ewes compared to E−/E-; however, total placentome number and caruncle weight did not differ due to ergot alkaloid treatment (Table 1). Exposure to ergot alkaloids during gestation reduced (*P* < 0.05) cotyledon weight by 34% compared to E−/E-. Fetal weight was 20% lower (*P* < 0.05) for E+/E+ ewes compared to E−/E-. Ratios of placental efficiency did not differ between ergot alkaloid treatments.

**Table 1:** Placental and fetal weights at d 133 of gestation for ewes fed endophyte-infected, ergot alkaloid containing (E+/E+) or endophyte-free, no ergot alkaloids (E−/E-) tall fescue seed during mid and late gestation

	E−/E-	E+/E+	SEM	P-Level
**Ewe**	4	4		
**Fetal weight, g**	9366.7	7440.4	316.47	0.0051
**Placentome weight, g**	922.1	649.4	51.05	0.0092
**Cotyledon weight, g**	705.3	467.7	56.47	0.025
**Caruncle weight, g**	216.7	181.7	13.03	0.106
**Placentome number**	98.25	112.5	12.18	0.44
**Fetal:Placentome weight ratio**	10.28	11.58	0.77	0.27
**Fetal:Caruncle weight ratio**	43.78	41.76	3.87	0.72
**Fetal:Cotyledon weight ratio**	13.54	16.49	1.56	0.23

### PCoA and RNAseq data

**Fig. 1:**
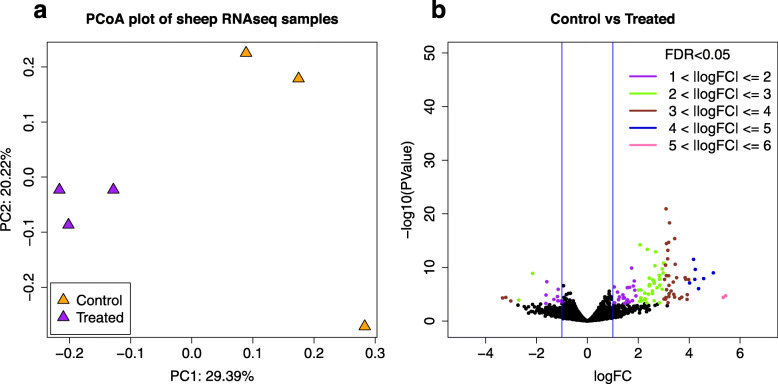
Principle Coordinates Analysis (PCoA) plot for RNAseq results in cotyledon tissues for ewes on E+/E+ fescue treatment compared to E−/E- at d 133 of gestation (**a**) and volcano plot for all DEGs detected in cotyledon tissues for ewes on E+/E+ fescue treatment compared to E−/E- at d 133 of gestation (**b**). Black dots represent an FDR ≥ 0.10 or |log_2_FC| < 1. Colored dots represent genes determined to be differentially expressed with a |log_2_FC| > 1 and an FDR < 0.10. Differences in color represent varying degrees of FC

A Principle Coordinates Analysis (PCoA) was conducted to visualize variation in the samples (Fig. 1). Principle component 1 (PC1) accounted for 29.39% of the variation in the samples and separated the samples based on fescue treatment. Principle component 2 (PC2) accounted for 20.22% of the variation. PC2 accounts for variation in the control samples whereas the E+/E+ samples remain closely associated with one another. In total, 13,572 genes were identified through mapping to the ovine genome across all samples (Table 2). Out of these, 110 genes were upregulated (FDR < 0.1) with 15 having a Log_2_FC between 0 and 1. The remaining 95 upregulated genes had a Log_2_FC ≥ 1. A total of 33 genes that were downregulated with 17 having a Log_2_FC between 0 and − 1. The remaining 16 downregulated genes had a Log_2_FC ≤ − 1. A volcano plot for all DEGs detected in cotyledon tissues for ewes on E+/E+ fescue treatment compared to E−/E- is presented in Fig. 1. Black dots represent an FDR ≥ 0.10 or |log_2_FC| < 1 while colored dots represent genes determined to be differentially expressed with a |log_2_FC| ≥ 1 and an FDR < 0.10.

**Table 2:** Summary of reads mapped to the ovine genome

	E−/E- Fescue Treatment			E+/E+ Fescue Treatment		
Sample	C1	C2	C3	T1	T2	T3
**Raw Reads**	10,576,330	10,770,727	8,425,934	10,988,240	9,267,649	10,034,493
**Clean Reads**	8,656,393	8,696,340	7,199,597	9,402,716	8,308,713	8,386,543
**Total Mapped**^a^	6,737,569	6,728,913	6,737,780	6,743,454	6,744,853	6,737,461
**Mapped**^b^**%**	98.29	98.16	98.29	98.37	98.40	98.29
**Annotated**^c^, %	43.26	43.38	42.65	44.74	45.15	43.95

^a^ Total mapped reads after downsampling

^b^Includes reads that aligned to the ovine reference genome. In the case of multimapped reads, each read was counted once

^c^Includes reads that were aligned to annotated genes in the reference genome

### Genes of interest

Genes of interest with an FDR < 0.01 and |log_2_FC| > 3 are presented in Table 3. Four genes had a |log_2_FC| > 5 in the cotyledon for ewes consuming E+/E+ treatment compared to E−/E- treatment. These included leukocyte cell derived chemotaxin 2 (*LECT2*), solute carrier family 22 member 9 (*SLC22A9*), apolipoprotein C3 (*APOC3*), and mannose binding lectin 2 (*MBL2*). In some cases, multiple genes within a given gene family were identified as being upregulated in the cotyledon tissue of E+/E+ treated ewes compared to E−/E- treatment. Five apolipoproteins (APOs) were upregulated including *APOC3*, *APOC4*, *APOA1*, *APOB*, and *APOH*. Several serpin (SERPIN) genes were also identified including *SERPINA3–8*, *SERPINF2*, *SERPINA1*, and *SERPIND1*. Inter-alpha-trypsin inhibitor heavy chains 1–3 (*ITIH1, ITIH2, ITIH3*) and the cytochrome P450 gene, *CYP2E1*, were also found to be upregulated in E+/E+ treatment compared to the control.

**Table 3:** Candidate genes from cotyledon tissues for ewes on E+/E+ fescue treatment compared to E−/E- at d 133 of gestation. Only genes with a |log_2_FC| > 3 and a FDR < 0.01 are displayed

Gene Symbol	Gene Name	Log_**2**_ Fold Change	FDR
LECT2	leukocyte cell derived chemotaxin 2	6.96	1.15E-09
SLC22A9	solute carrier family 22 member 9	5.43	5.44E-03
APOC3	apolipoprotein C3	5.38	7.90E-10
MBL2	mannose binding lectin 2	5.34	4.96E-03
PCK1	phosphoenolpyruvate carboxykinase 1	4.83	1.36E-08
APOC4	apolipoprotein C4	4.53	7.58E-06
FMO1	flavin containing monooxygenase 1	4.13	2.10E-05
ACSM1	acyl-CoA synthetase medium chain family member 1	4.06	1.63E-04
FABP1	fatty acid binding protein 1	3.87	1.92E-04
SERPINA3–8	serpin A3–8	3.75	1.45E-06
FMO3	flavin containing monooxygenase 3	3.49	3.31E-06
CYP2E1	cytochrome P450, family 2, subfamily E, polypeptide 1	3.47	8.43E-04
SPP2	secreted phosphoprotein 2	3.39	2.95E-07
AMBP	alpha-1-microglobulin/bikunin precursor	3.33	5.21E-15
ADH1C	alcohol dehydrogenase 1C	3.28	3.64E-06
ITIH3	inter-alpha-trypsin inhibitor heavy chain 3	3.26	3.08E-09
PLG	plasminogen	3.19	7.84E-12
AGT	angiotensinogen	3.16	6.73E-06
HP-25	protein HP-25 homolog 1	3.14	5.44E-03
TTR	transthyretin	3.13	7.84E-12
SERPINF2	serpin family F member 2	3.11	1.43E-05
HPD	4-hydroxyphenylpyruvate dioxygenase	3.08	2.60E-04
HPX	hemopexin	3.08	3.08E-09
VTN	vitronectin	3.08	2.45E-19
C6	complement C6	3.07	1.30E-05
FGA	fibrinogen alpha chain	3.06	2.70E-06
PAH	phenylalanine hydroxylase	3.05	1.43E-05
APOH	apolipoprotein H	3.05	2.95E-07
ALB	albumin	3.05	7.06E-03
HP	haptoglobin	3.04	3.02E-04
CPS1	carbamoyl-phosphate synthase 1	3.01	1.45E-06
ORM1	orosomucoid 1	3.02	4.06E-07
STRA6	stimulated by retinoic acid 6	−3.05	8.80E-03

**Fig. 2:**
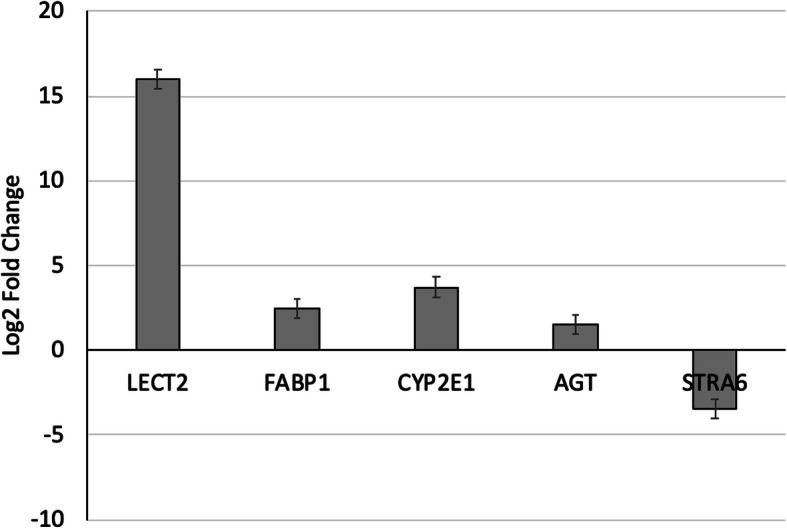
Differential expression of genes measured by qPCR in cotyledon of E+/E+ ewes compared to E−/E- (*n* = 4/treatment)

The five genes assessed using qPCR were found to be similar in their calculated Log_2_FC compared to RNA sequencing results (Fig. 2). The expression of *LECT2* and *CYP2E1* was found to be up-regulated (*P* < 0.05) in E+/E+ compared to E−/E-. Additionally, *STRA6* expression was down-regulated (*P* < 0.05) in E+/E+ compared to E−/E-. There was no statistical difference based on treatment for the two other genes tested, *AGT* and *FABP1*.

**Fig. 3:**
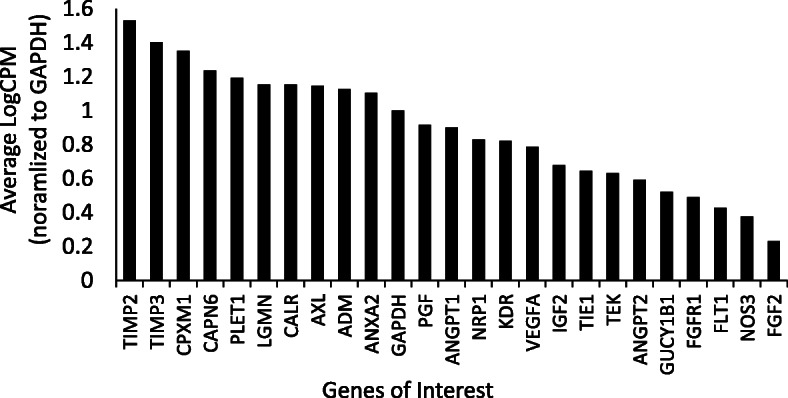
The average LogCPM normalized to GAPDH across all cotyledon samples regardless of treatment including vascular and growth factors and genes with high levels of expression that have not previously been identified in ovine cotyledon samples

Figure 3 shows the average LogCPM normalized to *GAPDH* for genes with high levels of expression that have not previously been identified in ovine cotyledon samples. These genes included TIMP metallopeptidase inhibitor 2 and 3 (*TIMP2*, *TIMP3*), carboxypeptidase X, M14 family member 1 (*CPXM1*), calpain 6 (*CAPN6*), and placentally expressed transcript 1 (*PLET1*).

### Gene ontology and KEGG pathway analysis

**Fig. 4:**
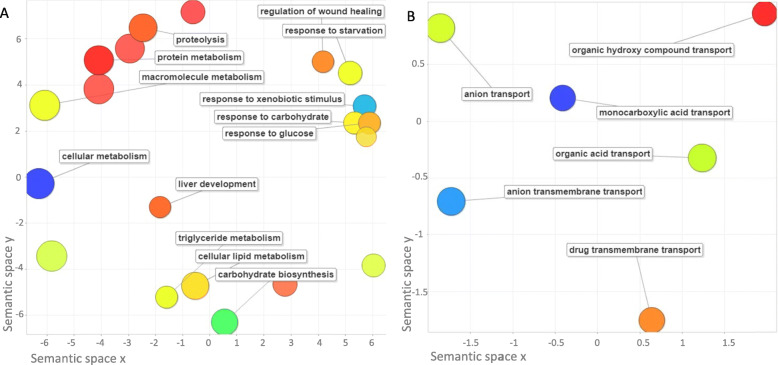
A scatterplot generated with REViGO shows a cluster representation of enriched biological process Gene Ontology (GO) term among upregulated genes (**a**) and downregulated genes (**b**)

REViGO generates a cluster representation of enriched biological process GO terms among upregulated genes. The REViGO analyses can be seen in Fig. 4 for upregulated and downregulated genes. A closer proximity denotes a closer relationship between the terms and an increase in size indicates fold-change. For upregulated genes, the most tightly associated cluster included responses to glucose, carbohydrates, and xenobiotic stimuli. There were two GO term clusters associated with lipid and protein metabolism. GO terms representing the response to starvation, regulation of wound healing, and liver development were also present but fairly independent of other terms. For downregulated genes, no clusters were present, but all enriched GO terms were associated with transport. This included anion, monocarboxylic and organic acid, and drug transmembrane transport. There was an enrichment for the regulation of peptide breakdown in E+/E+ cotyledon tissue compared to the control tissues. There was a fold enrichment of 15 or more for several types of peptidase and endopeptidase activity which included both regulation and inhibition of activity. The molecular functions of protease binding, carboxylic ester hydrolase activity, and carbohydrate derivative binding were also highly enriched in the gene set. As shown Table 4, KEGG pathway analysis identified two distinct pathways that were upregulated in E+/E+ treated samples. The complement and coagulation cascade was highly enriched and included plasminogen activator tissue type (*PLAT*), plasminogen (*PLG*), paraoxonase 3 (*PON3*), mannose binding lectin 2 (*MBL2*), complements *C9*, *C6*, and *C1S*, fibrinogen alpha chain (*FGA*), fibrinogen gamma chain (*FGG*), and serpins *SERPINF2*, *SERPINC1*, *SERPINA1* and *SERPIND1*. The peroxisome proliferator-activated receptor (PPAR) signaling pathway was also enriched and included apolipoprotein A2 (*APOA2*), apolipoprotein A5 (*APOA5*), apolipoprotein C3 (*APOC3*), enoyl-CoA hydratase and 3-hydroxyacyl CoA dehydrogenase (*EHHADH*), fatty acid binding protein 1 (*FABP1*), adiponectin (*ADIPOQ*), and phosphoenolpyruvate carboxykinase 1 (*PCK1*).

**Table 4:** KEGG pathway enrichment analysis using David (v 6.8) showed enrichment in the complement and coagulation cascade and PPAR signaling pathway for ewes on E+/E+ fescue compared to E−/E- treatment

Code	Pathway	FDR	Associated genes
Oas04610	Complement and coagulation cascades	2.44E-13	*PLAT, MBL2, C9, C6, C1S, PLG, FGG, FGA, SERPINF2, SERPINC1, SERPINA1, SERPIND1*
Oas03320	PPAR signaling	3.06E-03	*APOA2, EHHADH, APOC3, APOA5, FABP1, ADIPOQ, PCK1*

## Discussion

Exposure to ergot alkaloids during mid and late gestation reduced placentome and fetal weights at near-term, d 133 of an average 145 d gestation length. The reductions in the placentome weight were a direct result of reductions in the cotyledon, the fetal side of the placentome, mass and not of the caruncle, the maternal side. Estimates of placental sufficiency showed no difference in the fetal to cotyledon weight ratio, which suggests that the reduction in cotyledon weight limited growth of the fetus. During the first half of gestation, the ovine placenta experiences rapid proliferative growth and peaks in weight around d 80 after which a period of remodeling begins where connective tissue in the core of the fetal villi is replaced by vascular beds [31, 32]. This angiogenic vascular development in the cotyledon results in a 12-fold increase in capillary density, a decrease in capillary size, and continues throughout late gestation to support rapid fetal growth [33]. Previous research documents the extensive list of vascular and growth factors necessary during this process throughout mid and late gestation [15, 32–35] and differences in gene expression have been reported in cases of undernutrition [36], hyperthermia [37], and hypoxia [38]. Ergot alkaloids are known vasoconstrictors [39, 40] that bind to 5HT_2A_ serotonergic receptors. Previous examinations of known vascular factors involved in placental growth did not reveal any differences due to ergot alkaloid exposure [6] and therefore this RNA-sequencing project was developed to identify differentially expressed genes associated with ergot alkaloid exposure and discover potential adaptive mechanisms that alter nutrient supply to fetus.

The RNA-Seq results identified 95 genes that were up-regulated and 16 down-regulated in response to feeding mycotoxins. Interestingly, *LECT2* was the most upregulated gene with a 7-fold increase in the cotyledon samples of E+/E+ treated ewes. It is described as an energy-sensing hepatokine that is preferentially expressed in human adult and fetal liver cells but is secreted into the bloodstream [41]. Several studies have associated *LECT2* with glucose metabolism, insulin resistance and inflammation [42–44]. Knockout mouse studies show that loss of *LECT2* increases insulin signaling in skeletal muscle [41]. To our knowledge, *LECT2* expression has not been previously identified in placental tissues nor associated with IUGR. Therefore, further research is warranted to investigate the role of *LECT2* in cases of mycotoxin exposure and irregular placental development.

Ewes on E+/E+ fescue seed treatment during gestation experienced an upregulation in genes associated with responses to carbohydrates, glucose and carbohydrate biosynthesis. The main carbohydrate utilized by the placenta is glucose which is transported by GLUT transporters through the process of facilitated diffusion [45]. Under normal conditions the majority of glucose is derived from maternal circulation, but glucose can be derived from fetal blood if maternal concentrations are lacking [46]. Because of dependence on the concentration gradient, changes in the placental uptake and consumption of glucose is directly related to changes in maternal concentrations [47]. The upregulation in genes associated with responses to carbohydrates and glucose may indicate an increase in maternal concentrations of glucose reaching the placenta. However, plasma glucose concentrations were not different based on fescue seed treatment in the associated study [6]. During late gestation, placental consumption of glucose is up to 10 times higher than the fetus for the production of ATP and other sugars and carbohydrates including polyols [48]. The polyol pathways are highly active in the early placenta and are closely associated with the pentose phosphate pathway, which supports rapid cell proliferation [49, 50]. In sheep, additional carbohydrates such as lactate and fructose are produced and shuttled to umbilical and uterine circulations. Sheep with growth restricted placentae exhibit a reduction in overall glucose consumption on a placental weight basis, but an increase in the conversion of glucose (or other substrates) to lactate, much of which is shuttled to the fetus [51]. A similar scenario is seen in humans in which up to 22% of glucose is converted to lactate under normoxic conditions [52]. Because lactate is unable to be used by the placenta, it is speculated that this may be an attempt to set aside resources for the fetus [49]. The increase in carbohydrate biosynthesis suggests a similar scenario in which glucose or other substrates are converted to lactate or fructose at an increased rate in ewes on E+/E+ fescue seed treatment.

Triglyceride and cellular lipid metabolism were also upregulated in E+/E+ cotyledon samples compared to controls. Lipids and free fatty acids are required for growth and development of both the fetus and placenta but transport across the placenta appears minimal for most livestock species [53]. In sheep, the placenta may hydrolyze esterified lipids and then desaturate and/or elongate them to supply essential fatty acids to the fetus [53]. These processes, along with the synthesis of lipids from glucose and ketoacids, may provide necessary fats for both placental and fetal tissues during gestation [45]. Research also shows that volatile fatty acids (VFAs) produced through the process of rumen fermentation are utilized by the placenta and transported to the fetus in both cows and sheep [53]. It is hypothesized that the placental utilization of VFAs may work to generate ATP or synthesize fatty acids and, in adverse conditions, lipids could be utilized as an alternate energy source if insufficient glucose is available [48, 54]. In cases of maternal undernutrition in sheep, there is an increase in placental fatty acid transporters [55] and placental lipid metabolism is known to be altered in cases of IUGR [56]. Additionally, KEGG pathway analysis determined that PPAR signaling was upregulated in the cotyledon of E+/E+ treated ewes. Recent research has elucidated critical roles for PPARs in placental development and the pathophysiology of IUGR. PPARs are ligand-activated transcription factors that regulate gene expression in a variety of tissues and play a critical role in placental lipid metabolism [57]. Human placentae were found to have increased PPAR expression levels in cases of preeclampsia and IUGR when compared to controls and it has been suggested this may be an adaptive response to compensate for insufficient placental development [58]. *APOC3* is found within the PPAR pathway and had a 5-fold greater expression in cotyledon samples from E+/E+ treated ewes. *APOC3* is associated with hypertriglyceridemia and elevates plasma triglyceride levels by preventing clearance of very-low-density lipoproteins (VLDLs) and high-density lipoproteins (HDLs) but limited research is available denoting its presence or role in placental function.

A primary function of the placenta is to facilitate nutrient exchange to the fetus through the use of transport systems. Transporters are in place to mediate the transfer of endogenous compounds across the placental barrier but, in some cases, transporters accept specific exogenous substrates such as drugs or xenobiotics if they are present [59]. Such transporters are identified as drug transporters and include monocarboxylate transporters (MCTs) and anion transporters [59]. MCTs generally work to transport lactate, much of which is produced by placental metabolism when glucose is in short supply [60]. The placenta also uses anion transporters, such as organic anion transporters (OATs), which have been discovered in almost all barrier epithelia within the body [61]. Both MCTs and OATs also function to transport a wide range of drugs and toxins in addition to their endogenous substrates [59]. Ewes on E+/E+ fescue seed treatment experienced a consistent downregulation in a variety of transport systems, including monocarboxylic acid and anion transport, within the cotyledon compared to ewes on the control treatment. In contrast, solute carrier family 22 member 9 (*SLC22A9*; also known as *OAT7*) experienced a 5-fold increase in the cotyledon from E+/E+ ewes and was previously thought to be liver-specific [62]. It has been hypothesized that ergot alkaloids or metabolites may cross the placental barrier [7] but no research is available documenting the presence of these compounds in the fetus or which transport systems may be involved if placental transport exists. It was previously proposed that the downregulation of certain transport systems may augment fetal exposure to environmental toxicants during pregnancy [63]. A similar mechanism may be at work to limit fetal exposure to ergot alkaloids if placental transport occurs.

During late gestation, the primary function of the placenta is to facilitate nutrient exchange to the fetus in order to keep pace with exponential fetal growth [33]. REViGO revealed several upregulated GO clusters associated with macromolecule metabolism, including protein and lipid metabolism, in the cotyledon of ewes exposed to E+ fescue seed during gestation compared to those ewes on E- treatment. In addition to facilitating nutrient exchange to the fetus, the placenta is a metabolically active organ with specific energy and nutrient requirements. The placenta also utilizes, produces, and converts amino acids throughout gestation [53]. The placental protein turnover rate was previously reported at 60% per day in sheep [64]. Such high protein turnover rates are heavily dependent on the amino-acid availability in maternal circulation. During the initial period of placental growth in humans, the placenta is the primary recipient of maternally circulating amino acids. As fetal growth accelerates during late gestation, the fetus becomes the primary beneficiary of amino acids [65]. In sheep, the placenta is a net consumer of glutamate, serine, valine, leucine and isoleucine and shuttles greater concentrations of glutamine, methionine and glycine towards the fetus compared to levels found in maternal uterine circulation suggesting significant utilization and/or conversion of amino acids [66]. The placenta experiences a high level of protein synthesis during late gestation due to the changes in placental morphology. It was hypothesized that this period would result in a high rate of protein turnover [45, 67]. Nutritional stress in sheep has been shown to alter fetoplacental amino acid metabolism and cycling and in some cases changes in metabolism appear permanent [68, 69]. The upregulation in protein metabolism and proteolysis in the cotyledon of E+/E+ fescue treated ewes may indicate increased placental remodeling or a higher rate of protein turnover.

The KEGG pathway analysis determined the complement and coagulation cascade pathway was highly upregulated in the cotyledon of E+/E+ treated ewes. While the complement and coagulation networks are distinct, several key crossover points keep them linked and both must be tightly regulated during pregnancy. Increased thrombin production, which has been associated with preeclampsia and IUGR, may be a result of increased activation of the coagulation cascade beyond that of normal pregnancy [70]. Additionally, several coagulation components are necessary for vascular differentiation which is often impaired in the placentae of preeclamptic women [70]. Regulation of the innate and adaptive immune response through the complement system is necessary for successful placental and fetal development [71]. While some degree of activation is required, early embryonic loss and IUGR are often associated with increased complement activation [72]. The complement system has three routes of activation: the classical pathway, alternate pathway, and mannose-binding lectin pathway. The cotyledon samples of ewes exposed to E+/E+ fescue seed treatment had a 5-fold upregulation of *MBL*. Mannose binding lectin functions as a critical part of the innate immune system and works as a first line of defense against microorganisms [72]. Under normal physiological conditions, MBL does not recognize an organism’s own tissues. However, in cases of cellular hypoxia, altered cell surface glycosylation can stimulate *MBL* expression and activate the complement system [73]. In this study, ergot alkaloid induced vasoconstriction may result in placental hypoxia that stimulates MBL and the complement cascade.

Several genes presented with a high rate of expression that have not previously been identified in ovine cotyledonary tissues. *TIMP2* and *TIMP3* are members of the TIMP metallopeptidase inhibitor family that are involved in degradation of extracellular matrix and suppress endothelial cell proliferation. *CPXM1* is a member of the carboxypeptidase X, M14 family and placental expression has not been reported previously but it has been shown to be a positive regulator of adipogenesis [74]. *CAPN6* is a member of the calpain family which are calcium-dependent cysteine proteases shown to be highly expressed in the placenta where they are involved in tumorigenesis by promoting angiogenesis [75]. Placentally expressed transcript 1 (*PLET1*) has been characterized in the pig where it was shown to be involved in trophoblastic elongation of the conceptus [76]. While these genes did not show differences based on treatment, their high level of expression not previously reported in sheep was worth noting.

The research presented here was our initial exploration to identify the genes associated with reduced cotyledon mass observed when feeding mycotoxins containing ergovaline and ergovalinine. Using RNA-Seq methods, we identified several genes (*LECT2*, *SLC22A9*, *APOC3*, *MBL2*) that were highly (log_2_FC > 5; FDR < 0.01) up-regulated in response to mycotoxin exposure and one (*STRA6*) that was down-regulated (log_2_FC > 3; FDR < 0.01). Comparisons between RNA-Seq and qPCR showed 86% agreement in fold-change but genes with lower fold-change were not significant for qPCR. These comparisons are similar to those reported by Everaert and co-workers [77] who reported 85% agreement between RNA-Seq and qPCR comparisons of gene expression fold-change but genes with lower expression had inconsistent expression measurements. We acknowledge that this study had a very small sample size (*n* = 3 biological replicates per treatment) and that this is a limitation of the study. These results identified novel genes that may be involved in mycotoxin exposure and further research with larger numbers is warranted to examine how the cotyledon tissue responds to ergot alkaloid induced vasoconstriction.

## Conclusion

The present study demonstrates that the cotyledon transcriptome was altered in response to ergot alkaloid exposure during mid and late gestation compared to control ewes. Upregulated genes were in clusters for glucose, carbohydrates, lipid, protein, macromolecular and cellular metabolism, regulation of wound healing and response to starvation. For downregulated genes, no clusters were present, but all enriched GO terms were associated with anion and monocarboxylic acid transport. The complement and coagulation cascade and the peroxisome proliferator-activated receptor signaling pathway were found to be enriched for ewes consuming E+/E+ treatment. These results show that ergot alkaloid exposure upregulates genes involved in nutrient metabolism to supply the fetus with additional substrates in attempts to rescue fetal growth.

## Methods

### Experimental design

Placental samples utilized in this study were collected from a larger study and detailed experimental design information is available [6]. Mature Suffolk ewes (*n* = 57; 4.8 yr of age; 82 kg avg. BW), naïve to endophyte-infected tall fescue, were purchased from a private farm and transported to Clemson University Small Ruminant Facility 90 d prior to the start of the experiment. Ewes were synchronized using an intravaginal controlled internal drug release (CIDR) insert (Eazi-Breed CIDR, Zoetis Animal Health) for 7 d. Upon CIDR removal, ewes were given prostaglandin F2α (12.5 mg i.m.; Lutalyse; Pfizer, New York, NY) and turned in with a purebred Suffolk ram. The ram was fitted with a marking harness and crayon that was changed weekly. Ewes were checked twice daily for marks to estimate breeding date and confirmed pregnant by transrectal ultrasonography on d 30 of gestation. Pregnant Suffolk ewes (*n* = 32; *n* = 8/treatment) were randomly assigned to E+ (endophyte-infected; 1.77 mg/head/day of ergovaline and ergovalinine) or E- (endophyte-free; 0 mg/head/day of ergovaline and ergovalinine) tall fescue seed during MID (d 35–85) and/or LATE (d 86–133) gestation which provided four dietary treatments: E−/E-, E+/E-, E−/E+, and E+/E+. Ewes were individually penned into stalls (1.8 × 0.5 × 0.91 m) after 0700 and individually fed their respective treatment diet for 90 min. After individual feeding, ewes were removed from stalls and kept in a large pen (10–12 hd/pen) with ad libitum access to water and minerals (Purina Sheep Mineral, Land O’Lakes Inc., Arden Hills, MN), and with access to inside and outside areas devoid of forage or hay. For this study, a subsample (*n* = 4/treatment) was selected from the E−/E- and E+/E+ treatments that had twin fetuses and were representative of treatment means for further examination of cotyledon by RNA-seq. Ewe was the experimental unit for this study.

### Sample collection

On d 133 of gestation, ewes were transported to Godley-Snell Research Facility (14.3 km) at 0730 for terminal surgery. Each ewe was given an intravenous injection of Ketamine (10 mg/kg) and Diazepam (0.25 mg/kg) upon arrival for sedation and intubation. Ewes were intubated with a 10 mm endotracheal tube and placed on 4–5% isoflurane with 1–2 L/min of O_2_ for induction. Upon successful anesthetization, ewes were maintained at 3–5% isoflurane with 1–2 L/min of O_2_ and placed on a ventilator at 15–20 breaths per minute. Corneal reflex and heart rate were monitored to confirm adequate levels of anesthesia. The abdominal area was shaved, scrubbed with chlorhexidine, and ewes were subjected to a mid-ventral laparotomy. Once the uterus was exposed, an incision was made in the uterine wall to collect fetuses and a placentome of the type B morphology [78] was selected adjacent to the initial incision. Placentomes were immediately separated into the cotyledon and caruncle portions, and the cotyledon was flash frozen in liquid nitrogen and stored at − 80 °C for subsequent RNA extraction. Once removed, fetal lambs were euthanized with a 3 mL intracardiac injection of Beuthanasia-D Special (Merck Animal Health, Madison, New Jersey). Each fetus was towel dried and fetal weight was collected. Once all fetuses were removed, ewes were euthanized with an intravenous injection of 20 mL Beuthanasia-D Special. Placental and fetal data were analyzed using ANOVA with fescue treatment in the model.

### Sample preparation and RNA sequencing analysis

Total cellular RNA was extracted using Trizol reagent and purified using the PureLink Mini RNA kit (Invitrogen, Thermo Fisher Scientific, Waltham, MA) according to manufacturer instructions. RNA quantity was estimated using a NanoDrop 1000 Spectrophotometer (ThermoFisher). RNA integrity was determined using an Agilent 2100 Bioanalyzer (Agilent, Santa Clara, CA). All samples submitted for RNAseq had a RIN value of 9.3 or greater. RNA samples were shipped on dry ice to LCSciences (Houston, TX) for mRNA sequencing. Library preparation was completed using the Illumina Small RNA Sample Preparation Kit (Illumina, San Diego, CA USA). Samples were sequenced to an average sequencing depth of 10 M reads using an Illuminia HiSeq 2500 sequencing platform at 50 bp single end reads. Downsampling to ensure uniform coverage between samples was done using seqtk v1.3-r106 (https://github.com/lh3/seqtk). FastQC v0.11.7 was used to check quality of the samples [79]. Any low-quality bases and adapter sequences were trimmed using Trimmomatic v0.38 [80]. After trimming, 84.8% of the total trimmed reads across all samples were aligned to the ovine reference genome (ftp://ftp.ensembl.org/pub/release-96/fasta/ovis_aries/) using GSNAP v2018-07-04 [81]. Subread’s feature Counts v1.6.2 software was utilized to count uniquely mapped reads that aligned to known genes in the reference genome [82]. Raw gene counts were input to edgeR v3.22.5 and genes with at least 4 samples having at least 1 count per million (cpm) were kept and these counts were normalized [83]. A principal coordinates analysis plot was constructed showing one sample from each treatment to be an outlier. Sequencing and mapping data can be seen in Table 1. These samples were removed leaving *n* = 3/treatment as seen in Fig. 1. Raw counts from the subset samples were rerun through edgeR, filtering out genes with less than 3 samples having a cpm of less than 1, and used to calculate differentially expressed genes (DEG) in E−/E- vs E+/E+. A volcano plot was generated from the data. Genes were considered differentially expressed if their false discovery rate (FDR) was < 0.05 and their |log_2_FC| was greater than or equal to 1.

### Gene ontology (GO) and KEGG pathway analysis

Gene ontology (GO) enrichment analysis was conducted using Panther 14.1 (http://pantherdb.org/). DEGs with a |log_2_FC| greater than or equal to 1 were used to analyze upregulated and downregulated gene sets separately. *Bos taurus* was utilized as a reference in the absence of the sheep genome. Panther GO-Slim was used to assess biological process, molecular function, and cellular component. Enriched GO terms were semantically clustered and visualized using REViGO. Upregulated and downregulated genes were analyzed separately. David (v 6.8) was used to determine KEGG pathway enrichment.

### RT-qPCR validation

Gene expression analysis was conducted using quantitative real-time RT-PCR methods according to Duckett and co-workers [84] to serve as a comparison for RNAseq results. Aliquots from the same RNA samples submitted for RNAseq were converted to cDNA using qScript cDNA SuperMix (Quanta Bio, Beverly, MA) according to manufacturer instructions. Real-time PCR was performed using a QuantStudio3 (Applied Biosystems, Thermo Fisher) and Perfecta (Quanta Bio, Beverly, MA) SYBR green according to the manufacturer’s directions. An initial hold of 2 min at 95 °C was followed by 40 cycles of 95 °C for 15 s and 60 °C for 30 s. Primers for leukocyte cell derived chemotaxin 2 (LECT2), fatty acid binding protein 1 (FABP1), angiotensinogen (AGT), cytochrome p450 2E1 (CYP2E1) and stimulated by retinoic acid 6 (STRA6) were designed using PrimerQuest (Integrated DNA Technologies, IDT, Coralville, IA) and used to compare to RNA-Seq. Glyceraldehyde 3-phosphate dehydrogenase (GAPDH), βactin (bACT), and cyclophilin (CYC) were tested for stability using RefFinder for the selection of the most stable housekeeping gene [85]. The most stable housekeeping gene, GAPDH, was used for normalization. Normalized CT values (ΔCT = CT, gene − CT, GAPDH) were calculated for each sample. The relative quantification was the ratio of the target gene to internal control genes using the ΔΔCT method. Normalized CT values were analyzed using an ANOVA with fescue treatment in the model. Genes in cotyledon tissue from ewes fed E+/E+ fescue were considered differentially expressed relative to control at a cut of *P* < 0.05 for qPCR.

## Data Availability

RNA-Seq data are published and freely available in Gene Expression Omnibus GEO, accession number GSE155975, https://www.ncbi.nlm.nih.gov/geo/query/acc.cgi?acc=GSE155975.

## Abbreviations

5HT2a: 5-hydroxytryptamine receptor 2A
ADIPOQ: adiponectin
APO: apolipoprotein
APOA2: apolipoprotein A2
APOA5: apolipoprotein A5
APOC3: apolipoprotein C3
APOC4: apolipoprotein C4
APOA1: apolipoprotein A1
APOB: apolipoprotein B
APOH: apolipoprotein H
ATP: Adenosine triphosphate
BW: body weight
C1S: Complement C1S
C6: Complement C6
C9: Complement C9
CPM: counts per million
CYP3A4: cytochrome P450 3A4
CYP2E1: cytochrome P450 2E1
DEG: differentially expressed genes
EHHADH: Enoyl-CoA Hydratase And 3-Hydroxyacyl CoA Dehydrogenase
FABP1: Fatty acid binding protein 1
FC: Fold change
FDR: false discovery rate
FGA: Fibrinogen Alpha Chain
FGG: Fibrinogen Gamma Chain
GAPDH: Glyceraldehyde 3-phosphate dehydrogenase
GO: gene ontology
IUGR: Intrauterine growth restriction
ITIH: Inter-alpha-trypsin inhibitor heavy chains
LECT2: leukocyte cell derived chemotaxin 2
mRNA: messanger RNA
MBL2: mannose binding lectin 2
MCT: monocarboxylate transporters
PON3: paraoxonase 3
OAT: organic anion transporters
PCK: Phosphoenolpyruvate Carboxykinase 1
PCoA: Principle Coordinates Analysis
PLAT: Plasminogen Activator Tissue Type
PLG: Plasminogen
PPAR: peroxisome proliferator-activated receptor
RIN: RNA integrity number
RNA: ribonucleic acid
RNAseq: RNA sequencing
SERPINA1: Serpin Family A Member 1
SERPINA3–8: Serpin Family A Member 3–8
SERPINC1: Serpin Family C Member 1
SERPIND1: Serpin Family D Member 1
SERPINF2: Serpin Family F Member 2
1SLC22A9: solute carrier family 22 member 9
TMR: total mixed ration

## References

[1] Stuedemann JA, Hoveland CS (1988). Fescue endophyte: history and impact on animal agriculture. J Prod Agric.

[2] Young CA, Charlton ND, Takach JE, Swoboda G, Trammell MA, Huhman DV, Hopkins AA (2014). Characterization of *Epichloe coenophiala* within the US: are all tall fescue endophytes created equal?. Front Chem.

[3] Hoveland CS, Schmidt SP, King CC, Odom JW, Clark EM, McGuire JA (1983). Steer performance and association of *Acremonium coenophialum* fungal endophyte on tall fescue pastures. Agron J.

[4] Peters CW, Grigsby KN, Aldrich CG, Paterson JA, Lipsey RJ, Kerley MS, Garner GB (1992). Performance, forage utilization, and ergovaline consumption by beef cows grazing endophyte fungus-infected tall fescue, endophyte fungus-free tall fescue, and orchardgrass pastures. J Anim Sci.

[5] Duckett SK, Andrae JG, Pratt SL (2014). Exposure to ergot alkaloids during gestation reduces fetal growth in sheep. Front Chem.

[6] Britt JL, Greene MA, Bridges WC, Klotz JL, Aiken GE, Andrae JG (2019). Ergot alkaloid exposure during gestation alters. I. Maternal characteristics and placental development of pregnant ewes1. J Anim Sci.

[7] Klotz JL, Britt JL, Miller MF, Snider MA, Aiken GE, Long NM (2019). Ergot alkaloid exposure during gestation alters: II. Uterine and umbilical artery vasoactivity1. J Anim Sci.

[8] Greene MA, Britt JL, Powell RR, Feltus FA, Bridges WC, Bruce T, et al. Ergot alkaloid exposure during gestation alters: 3. Fetal Growth, Muscle Fiber Development and miRNA Transcriptome. J Anim Sci. 2019;97(4):1874–90. 10.1093/jas/skz068. 10.1093/jas/skz153 PMC6606534 31051033

[9] Sharma D, Sharma P, Shastri S (2017). Genetic, metabolic and endocrine aspect of intrauterine growth restriction: an update. J Matern Fetal Neonatal Med.

[10] Froen JF, Gardosi JO, Thurmann A, Francis A, Stray-Pedersen B (2004). Restricted fetal growth in sudden intrauterine unexplained death. Acta Obstet Gynecol Scand.

[11] McIntire DD, Bloom SL, Casey BM, Leveno KJ (1999). Birth weight in relation to morbidity and mortality among newborn infants. N Engl J Med.

[12] Cohen E, Baerts W, van Bel F (2015). Brain-sparing in intrauterine growth restriction: considerations for the neonatologist. Neonatology..

[13] Morrison JL (2008). Sheep models of intrauterine growth restriction: fetal adaptations and consequences. Clin Exp Pharmacol Physiol.

[14] Reynolds LP, Borowicz PP, Vonnahme KA, Johnson ML, Grazul-Bilska AT, Wallace JM (2005). Animal models of placental angiogenesis. Placenta..

[15] Reynolds LP, Borowicz PP, Vonnahme KA, Johnson ML, Grazul-Bilska AT, Redmer DA (2005). Placental angiogenesis in sheep models of compromised pregnancy. J Physiol.

[16] Lang U, Baker RS, Braems G, Zygmunt M, Kunzel W, Clark KE (2003). Uterine blood flow--a determinant of fetal growth. Eur J Obstet Gynecol Reprod Biol.

[17] Marconi AM, Ronzoni S, Vailati S, Bozzetti P, Morabito A, Battaglia FC (2009). Neonatal morbidity and mortality in intrauterine growth restricted (IUGR) pregnancies is predicated upon prenatal diagnosis of clinical severity. Reprod Sci.

[18] Reynolds LP, Caton JS, Redmer DA, Grazul-Bilska AT, Vonnahme KA, Borowicz PP (2006). Evidence for altered placental blood flow and vascularity in compromised pregnancies. J Physiol.

[19] Wallace JM, Regnault TR, Limesand SW, Hay WW, Anthony RV (2005). Investigating the causes of low birth weight in contrasting ovine paradigms. J Physiol.

[20] Dyer DC (1993). Evidence that ergovaline acts on serotonin receptors. Life Sci.

[21] Klotz JL, Brown KR, Xue Y, Matthews JC, Boling JA, Burris WR (2012). Alterations in serotonin receptor-induced contractility of bovine lateral saphenous vein in cattle grazing endophyte-infected tall fescue. J Anim Sci.

[22] Klotz JL, Aiken GE, Johnson JM, Brown KR, Bush LP, Strickland JR (2013). Antagonism of lateral saphenous vein serotonin receptors from steers grazing endophyte-free, wild-type, or novel endophyte-infected tall fescue. J Anim Sci.

[23] Poole DH, Lyons SE, Poole RK, Poore MH. Ergot alkaloids induce vasoconstriction of bovine uterine and ovarian blood vessels. J Anim Sci. 2018;96(11):4812–22. 10.1093/jas/sky328. 10.1093/jas/sky328 PMC6247839 30102353

[24] Barry JS, Anthony RV (2008). The pregnant sheep as a model for human pregnancy. Theriogenology..

[25] Leiser R, Krebs C, Ebert B, Dantzer V (1997). Placental vascular corrosion cast studies: a comparison between ruminants and humans. Microsc Res Tech.

[26] Beede KA, Limesand SW, Petersen JL, Yates DT (2019). Real supermodels wear wool: summarizing the impact of the pregnant sheep as an animal model for adaptive fetal programming. Anim Front.

[27] Hay WW, Brown LD, Rozance PJ, Wesolowski SR, Limesand SW (2016). Challenges in nourishing the intrauterine growth-restricted foetus - lessons learned from studies in the intrauterine growth-restricted foetal sheep. Acta Paediatr.

[28] Mikheev AM, Nabekura T, Kaddoumi A, Bammler TK, Govindarajan R, Hebert MF (2008). Profiling gene expression in human placentae of different gestational ages: an OPRU network and UW SCOR study. Reprod Sci.

[29] Majewska M, Lipka A, Paukszto L, Jastrzebski JP, Myszczynski K, Gowkielewicz M (2017). Transcriptome profile of the human placenta. Funct Integr Genomics.

[30] Cox B, Leavey K, Nosi U, Wong F, Kingdom J (2015). Placental transcriptome in development and pathology: expression, function, and methods of analysis. Am J Obstet Gynecol.

[31] Stegeman HJ (1972). A study of the maturation of the placenta in sheep. Acta Morphol Neerl Scand.

[32] Borowicz PP, Arnold DR, Johnson ML, Grazul-Bilska AT, Redmer DA, Reynolds LP (2007). Placental growth throughout the last two thirds of pregnancy in sheep: vascular development and angiogenic factor expression. Biol Reprod.

[33] Reynolds LP, Borowicz PP, Caton JS, Vonnahme KA, Luther JS, Buchanan DS (2010). Uteroplacental vascular development and placental function: an update. Int J Dev Biol.

[34] Carr DJ, David AL, Aitken RP, Milne JS, Borowicz PP, Wallace JM (2016). Placental vascularity and markers of angiogenesis in relation to prenatal growth status in overnourished adolescent ewes. Placenta..

[35] Reynolds LP, Redmer DA (2001). Angiogenesis in the placenta. Biol Reprod.

[36] Redmer DA, Milne JS, Aitken RP, Johnson ML, Borowicz PP, Reynolds LP (2012). Decreasing maternal nutrient intake during the final third of pregnancy in previously overnourished adolescent sheep: effects on maternal nutrient partitioning and feto-placental development. Placenta..

[37] Regnault TR, Galan HL, Parker TA, Anthony RV. Placental development in normal and compromised pregnancies-- a review. Placenta. 2002;23 Suppl A:S119–29. 10.1053/plac.2002.0792 11978069

[38] Zhang S, Barker P, Botting KJ, Roberts CT, McMillan CM, McMillen IC, et al. Early restriction of placental growth results in placental structural and gene expression changes in late gestation independent of fetal hypoxemia. Physiol Rep. 2016;4(23):e13049. 10.14814/phy2.13049. 10.14814/phy2.13049 PMC5357827 27923976

[39] Strickland JR, Looper ML, Matthews JC, Rosenkrans CF, Flythe MD, Brown KR (2011). Board-invited review: St. Anthony’s fire in livestock: causes, mechanisms, and potential solutions. J Anim Sci.

[40] Klotz JL (2015). Activities and effects of ergot alkaloids on livestock physiology and production. Toxins (Basel).

[41] Yamagoe S, Mizuno S, Suzuki K (1998). Molecular cloning of human and bovine LECT2 having neutrophil chemotactic activity and its specific expression in the liver. Biochim Biophys Acta.

[42] Lan F, Misu H, Chikamoto K, Takayama H, Jujychi A, Nihri K (2014). LECT2 functions as a hepatokine that links obesity to skeletal muscle insulin resistance. Diabetes.

[43] Jung TW, Chung YH, Kim HC, El-Aty AMA, Jeong JH (2018). LECT2 promotes inflammation and insulin resistance in adipocytes via P38 pathways. J Mol Endocrinol.

[44] Yoo HJ, Hwang SY, Choi JH, Lee HJ, Chung HS, Seo JA (2017). Association of leukocyte cell-derived chemotaxin 2 (LECT2) with NAFLD, metabolic syndrome and atherosclerosis. PLoS One.

[45] Bell AW, Ehrhardt RA (2002). Regulation of placental nutrient transport and implications for fetal growth. Nutr Res Rev.

[46] Simmons MA, Battaglia FC, Meschia G (1979). Placental transfer of glucose. J Dev Physiol.

[47] Hay WW, Molina RA, DiGiacomo JE, Meschia G (1990). Model of placental glucose consumption and glucose transfer. Am J Phys.

[48] Vaughan OR, Fowden AL. Placental metabolism: substrate requirements and the response to stress. Reprod Domest Anim. 2016;51(Suppl 2):25–35. 10.1111/rda.12797. 10.1111/rda.12797 27762057

[49] Jauniaux E, Hempstock J, Teng C, Battaglia FC, Burton GJ (2005). Polyol concentrations in the fluid compartments of the human conceptus during the first trimester of pregnancy: maintenance of redox potential in a low oxygen environment. J Clin Endocrinol Metab.

[50] Burton GJ, Fowden AL (2015). The placenta: a multifaceted, transient organ. Philos Trans R Soc Lond Ser B Biol Sci.

[51] Owens JA, Falconer J, Robinson JS (1987). Effect of restriction of placental growth on fetal and utero-placental metabolism. J Dev Physiol.

[52] Schneider H (2000). Placental oxygen consumption. Part II: in vitro studies--a review. Placenta.

[53] Vaughan OR, Fowden AL (2016). Placental metabolism: substrate requirements and the response to stress. Reprod D0mest Anim.

[54] Christie WW, Noble RC (1982). Fatty acid biosynthesis in sheep placenta and maternal and fetal adipose tissue. Biol Neonate.

[55] Ma Y, Zhu MJ, Uthlaut AB, Nijland MJ, Nathanielsz PW, Hess BW (2011). Upregulation of growth signaling and nutrient transporters in cotyledons of early to mid-gestational nutrient restricted ewes. Placenta..

[56] Cetin I, Alvino G (2009). Intrauterine growth restriction: implications for placental metabolism and transport. A review. Placenta.

[57] Xu Y, Wang Q, Cook TJ, Knipp GT (2007). Effect of placental fatty acid metabolism and regulation by peroxisome proliferator activated receptor on pregnancy and fetal outcomes. J Pharm Sci.

[58] Holdsworth-Carson SJ, Lim R, Mitton A, Whitehead C, Rice GE, Permezel M (2010). Peroxisome proliferator-activated receptors are altered in pathologies of the human placenta: gestational diabetes mellitus, intrauterine growth restriction and preeclampsia. Placenta..

[59] Ganapathy V, Prasad PD (2005). Role of transporters in placental transfer of drugs. Toxicol Appl Pharmacol.

[60] Nagai A, Takebe K, Nio-Kobayashi J, Takahashi-Iwanaga H, Iwanaga T (2010). Cellular expression of the monocarboxylate transporter (MCT) family in the placenta of mice. Placenta..

[61] Nigam SK, Bush KT, Martovetsky G, Ahn SY, Liu HC, Richard E (2015). The organic anion transporter (OAT) family: a systems biology perspective. Physiol Rev.

[62] Jungst C, Klein K, Eloranta JJ, Kullak-Ublick GA (2012). Bile acids Downregulate the human hepatic organic anion transporter 7. J Hepatol.

[63] Fontes KN, Reginatto MW, Silva NL (2019). Dysregulation of placental ABC transporters in a murine model of malaria-induced preterm labor. Sci Rep.

[64] Young M (1982). Protein turnover rate in early life. Acta Paediatr Acad Sci Hung.

[65] Chien PF (1991). Investigations of protein metabolism in human pregnancy: the term foetus and placenta studied using stable isotope labelled amino-acids. Clin Nutr.

[66] Chung M, Teng C, Timmerman M, Meschia G, Battaglia FC (1998). Production and utilization of amino acids by ovine placenta in vivo. Am J Phys.

[67] Vaughan OR, Rosario FJ, Powell TL, Jansson T. Regulation of placental amino acid transport and fetal growth. In: Huckle WR, editor. Molecular biology of placental development and disease 2017. p. 217–21. 10.1016/bs.pmbts.2016.12.008 28110752

[68] Kwon H, Ford SP, Bazer FW, Spencer TE, Nathanielsz PW, Nijland MJ (2004). Maternal nutrient restriction reduces concentrations of amino acids and polyamines in ovine maternal and fetal plasma and fetal fluids. Biol Reprod.

[69] Liechty EA, Kelley J, Lemons JA (1991). Effect of fasting on uteroplacental amino acid metabolism in the pregnant sheep. Biol Neonate.

[70] Klaitman V, Beer-Wiesel R, Rafaeli T, Mazor M, Erez O. The role of the coagulation system in preterm parturition. 2013. In: Perterm Birth [Internet]. IntechOpen. Available from: https://www.intechopen.com/books/preterm-birth/the-role-of-the-coagulation-system-in-preterm-parturition.

[71] Regal JF, Gilbert JS, Burwick RM (2015). The complement system and adverse pregnancy outcomes. Mol Immunol.

[72] Takahashi K, Ezekowitz RA (2005). The role of the mannose-binding lectin in innate immunity. Clin Infect Dis.

[73] Collard CD, Vakeva A, Morrissey MA, Agah A, Rollins SA, Reenstra WR (2000). Complement activation after oxidative stress: role of the lectin complement pathway. Am J Pathol.

[74] Kim YH, Barclay JL, He J, Luo X, O’Neill HM, Keshvari S (2016). Identification of carboxypeptidase X (CPX)-1as a positivie regulator of adipogenesis. FASEB J.

[75] Rho SB, Byun HJ, Park SY, Chun T (2008). Calpain 6 supports tumorigenesis by inhibiting apoptosis and facilitating angiogenesis. Cancer Lett.

[76] Zhao SH, Tuggle CK (2003). Linkage mapping and expression analyses of a novel gene, placentally expressed transcript 1 (PLET1) in the pig. Anim Gen.

[77] Everaert C, Luypaert M, Maag JLV, Cheng QX, Dinger ME, Hellemans J, Mestdagh P. Benchmarking of RNA-sequencing analysis workflows using whole-transcriptome RT-qPCR expression data. Sci Rep 7:1559. Doi:10.1038/s41598-017-01617-3. 10.1038/s41598-017-01617-3 PMC5431503 28484260

[78] Vatnick I, Schoknecht PA, Darrigrand R, Bell AW (1991). Growth and metabolism of the placenta after unilateral fetectomy in twin pregnant ewes. J Dev Physiol.

[79] Andrews S. http://www.bioinformatics.babraham.ac.uk/projects/fastqc2010. Available from: http://www.bioinformatics.babraham.ac.uk/projects/fastqc.

[80] Bolger AM, Lohse M, Usadel B (2014). Trimmomatic: a flexible trimmer for Illumina sequence data. Bioinformatics..

[81] Wu TD, Nacu S (2010). Fast and SNP-tolerant detection of complex variants and splicing in short reads. Bioinformatics..

[82] Liao Y, Smyth GK, Shi W (2014). featureCounts: an efficient general purpose program for assigning sequence reads to genomic features. Bioinformatics..

[83] Robinson MD, McCarthy DJ, Smyth GK (2010). edgeR: a bioconductor package for differential expression analysis of digital gene expression data. Bioinformatics..

[84] Duckett SK, Furusho-Garcia I, Rico JE, McFadden JW (2019). Flaxseed oil or n-7 fatty acid-enhanced fish oil supplementation alters fatty acid composition, plasma insulin and serum ceramide concentrations, and gene expression in lambs. Lipids.

[85] Xie F, Xiao P, Chen D, Xu L, Zhang B. miRDeepFinder: a miRNA analysis tool for deep sequencing of plant small RNAs. Plant Mol Biol. 2012;80:75–84. 10.1007/s11103-012-9885-2 22290409

